# β-Cell Deletion of Hypoxia-Inducible Factor 1α (HIF-1α) Increases Pancreatic β-Cell Susceptibility to Streptozotocin

**DOI:** 10.3390/ijms252413451

**Published:** 2024-12-15

**Authors:** Josephine Yu, Amit Lalwani, Jenny E. Gunton

**Affiliations:** 1 Centre for Diabetes, Obesity and Endocrinology (CDOE), The Westmead Institute for Medical Research, The University of Sydney, Sydney, NSW 2145, Australia; 2 Faculty of Medicine and Health, The University of Sydney, Sydney, NSW 2145, Australia; 3 Diabetes and Transcription Factors Group, Garvan Institute of Medical Research, Sydney, NSW 2010, Australia; 4 Department of Diabetes and Endocrinology, Westmead Hospital, Sydney, NSW 2145, Australia

**Keywords:** type 1 diabetes, streptozotocin, hypoxia inducible factor, glucose, beta cells

## Abstract

Type 1 diabetes (T1D) is caused by the immune-mediated loss of pancreatic β-cells. Hypoxia-inducible factor 1α (HIF-1α) is a transcription factor which is crucial for cellular responses to low oxygen. Here, we investigate the role of β-cell HIF-1α in β-cell death and diabetes after exposure to multiple low-dose streptozotocin (MLDS). MDLS triggers auto-immunity in susceptible animal models, such as non-obese diabetic (NOD) mice. These experiments used a novel mouse model with β-cell-specific deletion of HIF-1α on a NOD background (BIN mice). Mice were given 20 mg/kg MLDS for 5 consecutive days. Following MLDS, 100% of BIN mice developed frank diabetes versus 33% of floxed-control (FC) littermates and 17% of NOD controls (*p* < 0.001). BIN mice had obvious loss of β-cell mass (*p* < 0.0001) and increased necrotic areas within islets (*p* < 0.001). To confirm that diabetes was T1D, adoptive transfers of splenocytes from diabetic BIN and FC mice were performed on NOD-SCID (Severe Combined ImmunoDeficiency) recipients. All mice receiving BIN-splenocytes developed frank diabetes, confirming that MLDS induced true T1D. Interestingly, diabetes developed significantly faster in BIN-adoptive transfer mice compared to mice which developed diabetes after receiving an FC-adoptive transfer. These studies demonstrate the importance of β-cell HIF-1α in the preservation of β-cell mass and avoidance of auto-immunity.

## 1. Introduction

Type 1 diabetes (T1D) is an auto-immune disorder caused by immune-mediated loss of pancreatic β-cells. This leads to loss of insulin production and elevated blood glucose levels. The incidence of T1D is increasing globally. According to the World Health Organization, in 2017, over 9 million people had T1D [1]. T1D confers high life-time risks of diabetes complications, which include retinopathy, nephropathy, neuropathy, and vascular disease, leading to shortened life-expectancy even with best available medical care [2,3]. The pathogenesis of T1D involves a complex interplay between genetic and environmental factors. Concordance for T1D in identical twins is only around 50%, indicating a significant role for environmental factors. These environmental factors include viral infections, vitamin D deficiency, and some other factors such as early-life exposure to cereals such as oats and rye [4,5]. It is thought that these may initiate or promote the immune response against β-cells, particularly in genetically predisposed individuals.

HIF-1α is a transcription factor which plays a crucial role in the response to low oxygen levels. HIF is a heterodimeric transcription factor consisting of an alpha subunit (e.g., HIF-1α, HIF-2α) and a beta-subunit (HIF-1β (also called ARNT), HIF-2β, etc.) [6,7,8,9]. Under normoxic conditions, HIF-1α protein is degraded very quickly. It is hydroxylated at two proline residues (402 and/or 564) and one asparagine residue (803 in humans, 813 in mice) [10,11,12]. Hydroxylation recruits the von Hippel–Lindau (VHL)-containing E3-ligase complex, which ubiquitinates HIF-1α and leads to its proteolytic degradation [13,14,15]. In hypoxic conditions, HIF-1α hydroxylation is inhibited and protein rapidly accumulates [9].

Elevated HIF-1α protein levels in immune cells can stimulate the production of cytokines, inflammation, and reactive oxygen species, which can impair β-cell functionality and may cause apoptosis [16]. In human islet cultures, elevated HIF-1α expression is associated with apoptosis and production of reactive oxygen species [17,18]. However, we have previously demonstrated that HIF-1α improves β-cell function in mice [19] and that β-cell-specific HIF-1α is important for the prevention of T1D development using a mouse model treated with coxsackieviruses [20]. Genetic deletion of β-cell HIF-1α impairs islet transplant outcomes [21], and, conversely, increasing HIF-1α with iron chelation improves transplant outcomes, including for human islets [21]. Thus, islet exposure to hypoxia is likely to account for the association between increased HIF-1α and islet apoptosis; more severe islet hypoxia would simultaneously increase HIF-1α and increase islet cell death.

The hyperglycaemia of diabetes impairs the cellular response to hypoxia by destabilizing HIF-1α protein and inhibiting its transcriptional function [22,23]. Therefore, in diabetes, there is often impaired HIF-1α signaling [9].

Following our identification of protective effects of β-cell HIF-1α in islet transplantation [19,21] and in resistance to diabetes following viral exposure [20], we tested whether β-cell HIF-1α is important for preventing β-cell death and diabetes after exposure to the β-cell toxin streptozotocin (STZ) [24]. The multiple low-dose STZ (MLDS) model causes relatively low-grade β-cell damage with loss of a proportion of β-cells. In susceptible animals, that can trigger β-cell auto-immunity [24]. In the present study, we assess the role of β-cell-specific HIF-1α in T1D induction using MLDS in non-obese diabetes (NOD) mice. The hypotheses are (1) that mice lacking β-cell-specific HIF-1α will have significantly worsened diabetes after MLDS and (2) that any diabetes induced by MLDS in β-cell-specific HIF-1α null (BIN) mice is auto-immune.

## 2. Results

### 2.1. Optimal Multiple Low-Dose Streptozotocin (MLDS) Dosing

**Figure 1:**
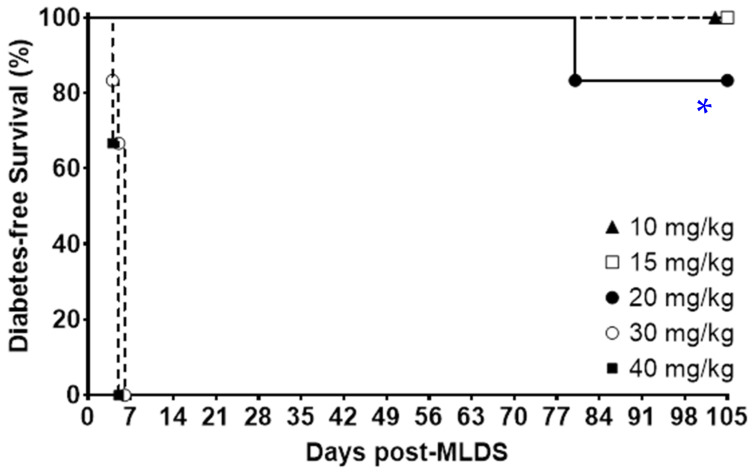
Dose optimization for MLDS. Kaplan–Meier curves for diabetes-free survival in NOD male mice treated with different doses of MLDS. *N* = 6 per dose. The 20 mg/kg dose was taken forward for the future studies because it did not induce any cases of immediate diabetes (as seen with 100% of the 30 mg/kg and 40 mg/kg mice). * *p* = 0.0002 using Mantel–Cox testing for 20 mg/kg versus 30 mg/kg or 40 mg/kg. *p* = not significant for 20 mg/kg versus 10 mg/kg or 15 mg/kg.

To identify the best dose for the MLDS protocol, male NOD mice were used, as they have only a 10% incidence of spontaneous diabetes by 6 months (after completion of these studies) [25]. Mice were 8 weeks old at the time of dosing. Mice were tested over 5 consecutive days with 10, 15, 20, 30 or 40 mg/kg of STZ (*N* = 6/group, 30 mice total). As shown in Figure 1, both 30 mg/kg and 40 mg/kg caused near-immediate diabetes, indicating that the higher doses cause direct β-cell death, as seen with ‘normal’ high-single-dose STZ, rather than inducing auto-immunity. Mice were considered to have florid diabetes when random-fed BGLs were >20 mmol/L on two occasions. No diabetes was seen with 10 mg/kg or 15 mg/kg in any control mice. Thus, further studies utilized 20 mg/kg MLDS to induce moderate β-cell damage without immediate β-cell death (*p* = 0.0002 for 20 mg/kg versus 30 mg/kg or 40 mg/kg, Mantel–Cox test).

### 2.2. BIN (Beta-Cell Specific HIF1α Null) Mice Have Increased Diabetes Incidence After MLDS

**Figure 2:**
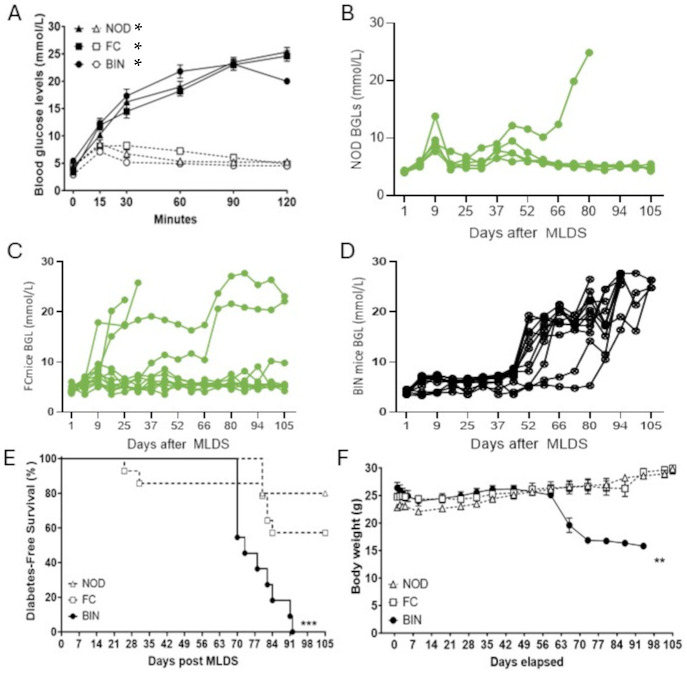
(**A**) Glucose tolerance tests (GTT) in mice. Baseline GTT (open shapes, broken line) in NOD (triangles, *N* = 5), FC (squares, *N* = 5), and BIN (circles, *N* = 6) mice and at day 10 post-MLDS (solid shapes and lines; NOD, *N* = 5; FC, *N* = 12, BIN, *N* = 11) * = *p* < 0.05 versus matching pre-MLDS mice. (**B**–**D**) Random-fed blood glucose levels (rBGL) after MLDS. Individual mouse monitoring of rBGL in NOD (**B**) (*N* = 5), (**C**) FC (*N* = 12), and (**D**) BIN (*N* = 11). (**E**) Diabetes-free survival in MLDS-treated mice. Significantly more BIN mice (100%; *N* = 11 of 11) developed diabetes by day 91, in comparison to NOD (17%; *N* = 1 of 5) and FC (33%; *N* = 4 of 12) counterparts. *** = *p* < 0.001 by Mantex–Cox survival testing. (**F**) Mean bodyweight over time. BIN (*N* = 11) mice had significantly reduced bodyweight compared to NOD (*N* = 5) and FC (*N* = 11) mice. All BIN mice were culled before day 98 due to diabetes, so there are no weights available after that point. ** *p* < 0.01 for BIN mice compared to controls (ANOVA for repeated measures (ANOVArm)).

Following the optimization of MLDS dosage, 8-week-old NOD, floxed-control (FC), and BIN mice were given 5 consecutive days of 20 mg/kg MLDS using intraperitoneal injection. Baseline glucose tolerance tests (GTTs) were conducted in all three groups and, as expected, baseline GTTs were normal (Figure 2A, broken lines). However, 10 days after MLDS, all mice experienced significantly impaired glucose tolerance (Figure 2A, solid lines, each *p* < 0.05 versus matched pre-MLDS mice). Note that the fasting blood glucose levels (BGLs) remain completely normal in all mice; no mouse had type 1 diabetes at that early timepoint.

After that, mice were followed until development of diabetes or for a total of 15 weeks. BGL monitoring (Figure 2B–D) shows that by day 91 post-MLDS, 17% of NOD mice (one out of five) and 33% of FC mice (four out of twelve) developed diabetes (Figure 2B,C). In comparison, 100% of BIN mice have developed diabetes (11 out of 11 mice, Figure 2D, *p* < 0.001 by Fisher’s exact test). Diabetes-free survival curves are shown in Figure 2E. In parallel with the development of diabetes, these BIN mice show significant weight loss, a classic symptom of florid diabetes (Figure 2F).

### 2.3. Decreased β-Cell Mass and Lower Serum Insulin in BIN Mice Post-MLDS

**Figure 3:**
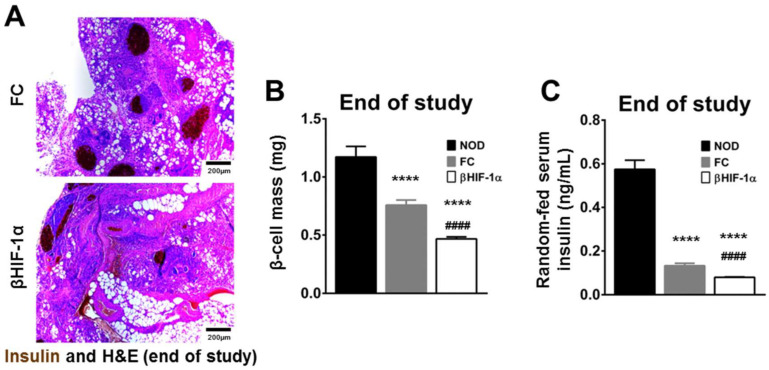
(**A**) Representative images of pancreatic sections from FC (**A**) and BIN (**B**) mice at the end of the study following MLDS injections. (**B**) Quantification of β-cell mass. Significant reduction in β-cell mass in FC (*N* = 8) compared to NOD (*N* = 6). BIN (*N* = 8) mice had significantly reduced β-cell mass compared to FC and NOD. (**C**) Serum insulin concentrations. Significant reduction in random-fed serum insulin concentrations were seen at the end of the study in FC (*N* = 8) and BIN (*N* = 8) mice vs. NOD (*N* = 6) counterparts. BIN mice had further reduced insulin concentration vs. FC mice. **** *p* < 0.0001 versus NOD. #### *p* < 0.0001 BIN versus FC, Student’s *t*-tests for the pair-comparisons.

At sacrifice, histological analyses of pancreatic sections show that β-cell mass is reduced in FC compared to NOD mice (0.76 ± 0.04 vs. 1.17 ± 0.09 mg; *p* < 0.0001, Figure 3A,B). However, the deterioration in β-cell mass (Figure 3C) is more severe in BIN mice, who were 38% lower than floxed-controls and 60% lower than NOD-controls (BIN mice 0.47 ± 0.02, *p* < 0.0001 vs. FC and vs. NOD).

Serum insulin analyses support these results (Figure 3C). A reduction in serum insulin is present in FC mice compared to NOD mice (0.13 ± 0.01 vs. 0.58 ± 0.04 ng/mL; *p* < 0.0001). However, βHIF-1α-null NOD mice show a stark reduction in insulin levels compared to both FC and NOD mice at 0.08 ± 0.002 ng/mL (both *p* < 0.005). The reduced insulin levels are inappropriate in the setting of the very elevated BGLs.

### 2.4. MLDS Induces Necrosis in Islets of BIN Mice

**Figure 4:**
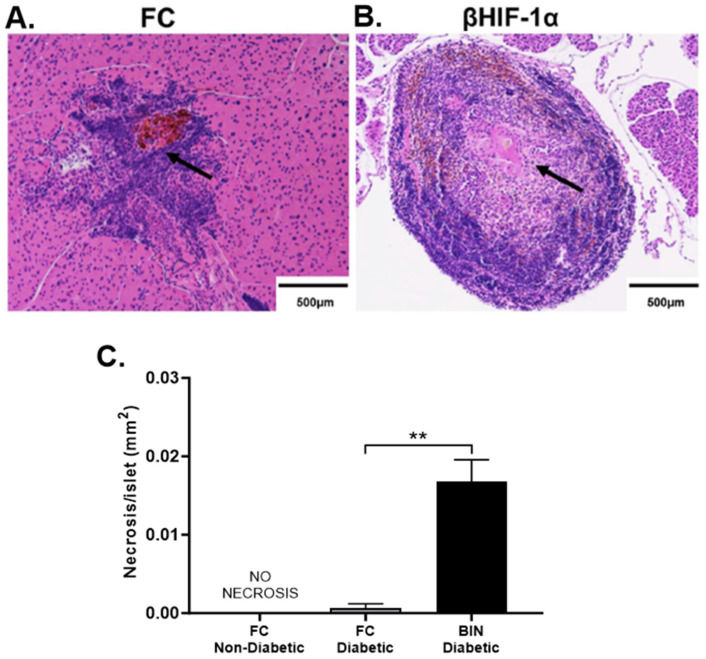
(**A**,**B**) Representative images of pancreatic islets from FC (**A**) and BIN (**B**) mice at the end of the study following MLDS injections. Necrosis shown in black arrows for FC and BIN mice. It was not observed in NOD mice. Scale bar equals 500 µm. (**C**) Quantification of necrotic area per islet. No necrosis detected in NOD mice (*N* = 0 of 6). Only two out of ten FC mice had necrosis in islets, in comparison to the four out of twelve BIN mice who had a significantly greater area involved. ** *p* < 0.01 BIN diabetic versus FC diabetic, Student’s *t*-test.

Pancreatic sections stained with insulin from FC (Figure 4A) and BIN (Figure 4B) mice show some areas of necrosis within islets at sacrifice compared to NOD controls (Figure 4C). Quantification of these sections shows a significant increase in necrotic area as a proportion of islet area in BIN mice post-MLDS (33%) compared to FC (20%; *p* = 0.0042) (Figure 4C). Interestingly, none of the NOD control mice have detectable areas of necrosis within islets.

### 2.5. Confirmation of Auto-Immune T1D in BIN Mice

Confirmation that diabetes is auto-immune is provided when adoptive transfer of immune cells from the diabetic subject to another, immunodeficient, non-diabetic mouse causes T1D in the recipient. Here, we performed adoptive transfer from the streptozotocin treated mice into NOD-SCID mice.

**Figure 5:**
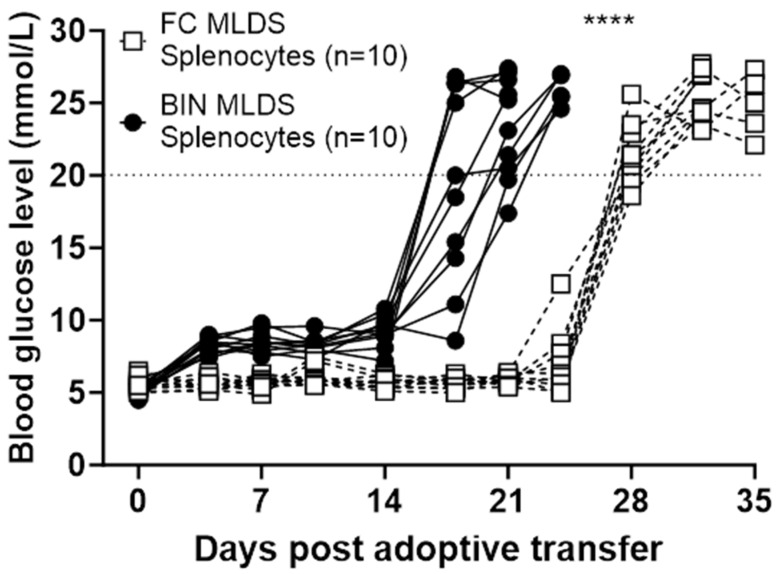
BGL monitoring following adoptive transfer of splenocytes into NOD-SCID recipient mice. Random-fed blood glucose concentrations in NOD-SCID recipients (*N* = 10 recipients/group) that received splenocytes from either diabetic MLDS FC mice (white squares) or diabetic MLDS BIN mice (dark circles). Mice receiving BIN-mice splenocytes developed diabetes by day 21, significantly earlier than mice receiving FC-mice splenocytes. **** *p* < 0.001, Mantel–Cox survival test.

Splenocytes were isolated and transferred into recipients using tail vein injection. By day 21 post-transfer (Figure 5), 100% of mice who received splenocytes from diabetic BIN mice (*N* = 10) develop florid diabetes. This confirms that the diabetes in BIN mice is auto-immune in nature.

In recipient mice who received splenocytes from FC controls and who developed diabetes after MLDS, there is also 100% diabetes incidence, confirming that their diabetes was also immune-mediated. Interestingly, this shows delayed onset at around 5 weeks after adoptive transfer, which is significantly delayed compared to BIN mice (*p* < 0.001), suggesting more efficient β-cell killing by T-cells from BIN diabetic mice.

## 3. Discussion

Despite the relative hypoxia which can be present in diabetic tissues [26], the stability of HIF-1α protein is compromised in people with diabetes, and also in pre-clinical models of diabetes [27,28,29]. That leads to reduced HIF1α protein in tissues in animal models of diabetes. Thus, the model here with reduced β-cell HIF1α is of relevance to diabetes.

A major hypothesis for the priming of auto-immunity in type 1 diabetes relies on an initial insult to β-cells, which leads to presentation of β-cell antigens to immune cells. That triggers auto-immune recognition of β-cells. There is strong evidence that viral infections can trigger this immunity, for example, the >10% incidence of T1D after congenital rubella exposure [30,31]. There is also a longstanding body of work supporting increased incidence of diabetes after exposure to coxsackieviruses [32,33]. Consistent with that, we previously reported that HIF-1α in β-cells plays a critical role in T1D development following coxsackievirus insult (CVB1 or CVB4) [20].

However, any cause of damage which facilitates the antigen presentation of β-cell antigens could theoretically prime auto-immunity. Here, we used the known β-cell toxin, streptozotocin to assess the role of β-cell HIF-1α in non-viral T1D development. At high doses, streptozotocin causes abrupt, large-scale β-cell death and insulin-deficient diabetes within 4–5 days, without requirement for any auto-immunity [34].

In contrast, multiple low-dose streptozotocin (MLDS) at the correct dose for the strain of mice triggers T1D in a proportion of previously non-diabetic NOD mice. In our studies, MLDS caused significant glucose intolerance without type 1 diabetes within 2 weeks of streptozotocin. Absence of T1D is shown by the completely normal fasting glucose levels in the absence of insulin treatment. The glucose results at 2 weeks were very similar in each set of mice, suggesting that there was no differential induction of β-cell death between groups. Despite these highly similar results at 2 weeks, the long-term diabetes outcomes were very different.

Normally, normal NOD mice have a low (approximately 11%) spontaneous T1D incidence by the age of 13 weeks [35]. MLDS induced short term T1D in 29% of control NOD mice within 15 weeks (5 of the 17 control mice). In contrast, in BIN mice, which lack β-cell HIF1α, there was 100% diabetes induction within 15 weeks of MLDS. This clearly demonstrates an important role for β-cell HIF-1α in resistance to T1D after a β-cell insult.

We and others have previously shown that HIF-1α has important effects on β-cell function [19]. Separately, the presence of β-cell HIF1α is required for resistance to β-cell death after the stress of islet transplantation [21]. Increasing the HIF1α protein by inhibiting its degradation improves islet transplant outcomes for mice and human islets [21]. The data presented here suggest the presence of HIF1α, also.

Consistent with the 100% diabetes incidence in BIN mice, we find a more severe loss of β-cell mass in BIN mice compared to FC and NOD controls. Of note, pancreatic islets in BIN mice also showed an interesting increase in islet necrotic area. Necrosis is also thought to be an important driver of β-cell death in ‘fulminant’ T1D [36]. Necrosis occurs with overwhelming insult to cells when the cell is too damaged to undergo the more orderly, less locally toxic apoptotic cell death.

It is important to highlight the key differences between MLDS and CVB4 models for the study of T1D. MLDS presents a more ‘direct’ auto-immune model, as there is no ongoing β-cell damage, which differs from an uncleared viral infection. Horwitz and colleagues [37] previously investigated the similarities between STZ and CVB4 in the development of T1D. They suggested that coxsackievirus accelerates β-cell damage rather than acting as an immune modulator [38,39,40]. The MLDS model causes the direct death of a proportion of β-cells, releasing β-cell auto-antigens. This primes the immune response in susceptible animals. Consistent with the diabetes after MLDS being auto-immune, transfer of splenocytes from mice with diabetes after MLDS induced diabetes in all NOD-SCID recipients.

Pancreatic β-cells are relatively ‘fragile’, and they are susceptible to induction of apoptosis after many different insults [41,42,43,44]. Stressed β-cells also release more improperly folded insulin, which induces the unfolded protein response [45] and endoplasmic reticulum stress [46]. Additionally, improperly folded insulin is thought, in many cases, to be the main auto-antigen, which triggers the anti-β-cell immune response [47]. Decreased β-cell HIF1α could contribute to type 1 diabetes pathogenesis by increasing sensitivity to apoptosis, increasing β-cell stress (as HIF1α mediates a protective response to stress), and by increasing the load of improperly folded insulin [48].

Overall, these data show that presence of HIF1α in pancreatic β-cells is important for avoiding T1D after exposure to a β-cell toxin. HIF1α is normally induced by inflammation, infection, and/or hypoxia. The increase in HIF1α protein enables its transcriptional activity, which induces a beneficial protective response to enhance cell survival. If the HIF1α induction is blocked, this would facilitate cell death, increased release of potential antigens, triggering of auto-immunity and development of T1D.

## 4. Materials and Methods

### 4.1. Animal Experiments

The present study used NOD mice purchased from the Animal Resource Centre (ARC, Canning Vale, WA, Australia). The β-cell HIF-1α mouse has homozygous floxed HIF-1α genes and is transgenic for Cre-recombinase driven by the rat insulin promoter (RIP-Cre) [19,21,49]. Thus, as previously published, in insulin expressing cells, RIP-Cre is expressed, and the floxed gene (in this case HIF-1α) is deleted. Mice that are homozygous for floxed HIF-1α but do not have the floxed gene are otherwise normal and were used as a control group; they are floxed-control (FC) mice. β-cell specific HIF-1α mice crossed with NOD mice to generate βHIF-1α NOD mice, called BIN from here [19,21,49]. This backcrossing was performed at the Australian BioResources (ABR, Moss Vale, NSW, Australia). At least 12 generations were crossed and all IDD loci tested were NOD, not B6. Experimental mice were housed in the Biological Services Facility (BSF) at the Westmead Institute for Medical Research and kept on a 12 h:12 h light–dark cycle. Mice were housed with a maximum of 5 mice per cage in individually ventilated cages kept at a constant temperature of 22 °C. All mice were given ad libitum access to a standard chow diet (Agrifood Technology, Australia), currently able to be sourced from Specialty Feeds as diet SF00-100. All procedures were approved by the Western Sydney Local Health District Animal Ethics Committee under protocol number # 4274, approved 1 June 2019. Some of these results presented here in detail were presented in text, without figures in [20].

Dose–response studies were performed in 8-week-old male NOD mice, aiming to achieve glucose intolerance without florid diabetes. We assessed 10, 15, 20, 30, and 40 mg/kg of repeated STZ injections for 5 consecutive days in NOD mice. All mice developed rapid diabetes with 30 and 40 mg/kg, suggesting direct β-cell death rather than auto-immunity. Thus, 20 mg/kg was used for these experiments.

Following the dose-optimizing experiment, 8-week-old male NOD mice, floxed-control (FC), and BIN mice were given intraperitoneal injections of STZ at 20 mg/kg for 5 consecutive days. Mice were monitored twice a week for bodyweight and blood glucose levels (BGLs). BGLs were assessed with the FreeStyle Lite glucometer (Abbott Diabetes Care, Macquarie Park, Australia), as previously reported [20]. Mice that had random-fed BGLs (rBGL) of ≥20.0 mmol/L on ≥2 days were considered diabetic. These studies were conducted in 2 cohorts. The first cohort had pre-MLDS and post-MLDS glucose tolerance testing, and the second cohort only had post-MLDS glucose tolerance testing, as the baseline levels were normal in the first cohort.

### 4.2. Glucose Monitoring and Glucose Tolerance Test (GTT)

Mice were fasted in the morning for 4 h prior to the GTT, which was performed in the afternoon. Then, mice were injected with 20% dextrose solution at 2 g/kg intraperitoneally. BGLs were measured at 0, 15, 30, 60, 90, and 120 min post-dextrose injection, as previously reported [20,49].

### 4.3. Histology

Paraffin-embedded tissues were sectioned and prepared for immunohistochemistry with haematoxylin and eosin (H&E) counterstaining. In each case, slides were incubated at 70 °C for 1 h prior to dewaxing.

Sections were immuno-stained for insulin using an autostainer (Dako Autostainer Plus Link, Dako, Santa Clara, CA, USA). The staining was performed using the DAKO kit, as per manufacturer’s protocol. Briefly, sections were dewaxed and rehydrated, then blocked for endogenous peroxidase activity. Slides were incubated with primary antibody for 1 h at room temperature (rabbit anti-insulin antibody; #4590, Cell Signaling Technology Inc., Danvers, MA, USA). Dako antibody diluent was used for all dilutions. Following a wash, secondary HRP-tagged anti-rabbit antibody (provided by the Dako staining kit) was used to incubate slides. After another wash, DAB and chromogen substrate buffer was used. H&E counterstaining used a standard H&E staining protocol was performed, as previously described [20,49,50], using the Leica autostainer XL (Leica Biosystems, Nussloch, Germany). Briefly, slides were incubated in hematoxylin followed by tap water bath. Slides were then exposed to acid alcohol and Scott’s blue briefly before staining with eosin. The slides were then placed into ethanol baths for dehydration and xylene baths for clearing before cover-slipping. Coverslips were mounted using non-aqueous mounting fluid (#MM24, Leica Biosystems, Germany) in an automated cover-slipping machine (Leica CV5030, Leica Biosystems, Mt Waverley, Australia).

Using the insulin immunohistochemistry with H&E counterstaining, necrosis (amorphous cell-free eosinophilic areas) was measured using imageJ and by ‘circling’ these areas.

### 4.4. Quantification of β-Cell Mass

β-cell mass was calculated by measuring β-cell area per area of whole sections of pancreas for every 20th section of the pancreas for every mouse, as previously described [20,50]. The “first” section was identified as the first section of pancreas to have positive staining. The islet area was measured using ImageJ 1.47 software (NIH freeware) and quantified as a percentage of whole pancreas area multiplied by the pancreatic weight to obtain β-cell mass.

### 4.5. Adoptive Transfer

For adoptive transfer, donor mice were sacrificed, and spleens were collected in phosphate-buffered saline (PBS, Merck, Bayswater, VIC, Australia. Cat P4474-500ML) containing 1% bovine calf serum (Sigma-Aldrich, Baywater, VIC, Australia. Cat 12133C-500ML). The donors were either BIN or floxed-controls, and had diabetes at the time of splenocyte collection. Splenocytes were dissociated using a syringe plunger over a 70 µm cell strainer and centrifuged twice at 435× *g* for 5 min at 4 °C. Cells were resuspended in sterile phosphate-buffered saline (PBS) before intravenous injection in recipient mice via the tail vein.

Recipient NOD mice with severe combined immunodeficiency (NOD-SCID) due to homozygous mutations in *Prkdc^scid^* were purchased from ARC or ABR animal facilities. Each recipient mouse received 2 × 10^7^ cells suspended in 200 µL of sterile saline. Recipient mice were weighed, and their blood glucose levels (BGLs) were checked at least twice per week.

### 4.6. Statistical Analyses

Data were analyzed using GraphPad Prism Version 10 (San Diego, CA, USA). Continuous variables were assessed for normality using the D’Agostino and Pearson omnibus normality test. Student’s unpaired *t*-test was used to compare two groups and ANOVA was used for three or more groups. Where there were serial assessments of the same measurement in the same animal, ANOVA for repeated measures (ANOVArm) was used. These assessments were corrected for by Tukey or Bonferonni’s post hoc tests. Kaplan–Meier curves were analyzed using Logrank (Mantel–Cox) test. All data are presented as mean ± SEM, unless otherwise specified.

## 5. Conclusions

MLDS induces T1D in a small proportion of diabetes-susceptible NOD mice. However, NOD mice with deleted HIF-1α in their β-cells had 100% diabetes incidence after MLDS. Together, our results show an important role for β-cell HIF-1α in the triggering of auto-immunity and β-cell survival.

## Data Availability

Data will be available on request from the corresponding author.
